# Dietary Fiber and Lysolecithin Supplementation in Growing Ducks: Effect on Performance, Immune Response, Intestinal Morphology and Lipid Metabolism-Regulating Genes

**DOI:** 10.3390/ani11102873

**Published:** 2021-09-30

**Authors:** Mohamed I. El-Katcha, Mosaad A. Soltan, Ramadan Shewita, Safaa E. Abdo, Amr S. Sanad, Vincenzo Tufarelli, Mahmoud Alagawany, Karima El-Naggar

**Affiliations:** 1 Nutrition and Veterinary Clinical Nutrition Department, Faculty of Veterinary Medicine, Alexandria University, Alexandria 22758, Egypt; mohamedelkatcha@ymail.com (M.I.E.-K.); mossad.sultan@alexu.edu.eg (M.A.S.); ramadan.saiid@alexu.edu.eg (R.S.); karima.muhammad@alexu.edu.eg (K.E.-N.); 2 Genetics and Genetic Engineering, Department of Animal Wealth Development, Faculty of Veterinary Medicine, Kafrelsheikh University, Kafrelsheikh 33516, Egypt; safaa_2m@yahoo.com; 3 Veterinarian, Faculty of Veterinary Medicine, Kafrelsheikh University, Kafrelsheikh 33516, Egypt; kem_8623@yahoo.com; 4 Department of DETO, Section of Veterinary Science and Animal Production, University of Bari ‘Aldo Moro’, 70010 Valenzano, Italy; 5 Department of Poultry, Faculty of Agriculture, Zagazig University, Zagazig 44511, Egypt

**Keywords:** ducks, crude fiber, soya hulls, lysolecithin, growth, lipid, immunity

## Abstract

**Simple Summary:**

Searching for and introducing unconventional feeds in ducks’ diets has become a major concern. However, low-priced feed ingredients such as rice bran and seed hulls are generally low in energy with high dietary fiber content. Thus, this study focused on the effects of different dietary fiber levels (with or without lysolecithin) on the performance, immune response, expression of some lipid regulating genes, and intestinal morphology of ducks. From our results, increasing fiber level in the diet (with or without the addition of lysolecithin) altered duck performance and intestinal morphology, improved immunity, and lowered serum lipid profile with a modulatory effect on the expression of lipid metabolism-regulating genes.

**Abstract:**

The impact of different dietary fiber (DF) levels (with or without lysolecithin supplementation) on growth performance, immune response, expression of some lipid regulating genes and intestinal morphology was assessed in 408 Pekin ducks for 2 months. Soybean hulls were added to the diet to provide four different levels of DF: 2.4 (control diet), 3.8, 5.3, and 6.7% for the first four groups, respectively, while groups 5 to 8 fed the same four levels of DF with lysolecithin addition. Increasing dietary DF non-significantly reduced (*p* > 0.05) the ducks’ body weight (BW). However, ducks fed on 3.8% DF showed higher BW and improved feed conversion ratio. Lysolecithin supplementation with different DF did not support growth performance. Increasing DF with or without lysolecithin had no effect on serum lipid profile (*p* > 0.05). However, serum high-density lipoproteins (HDL) concentration was significantly increased with increasing fiber level in diet (*p* ˂ 0.05). Increasing DF with or without lysolecithin addition increased serum antioxidant activities and improved the immune response in terms of phagocytic and lysozyme activities. The DF level reduced the duodenal villi length and mucosal layer thickness while increased the villi width (*p* ˂ 0.05). Lysolecithin supplementation to diets ameliorated adverse effects on intestinal morphology. Moreover, DF level in ducks’ diet with or without lysolecithin significantly upregulated the expression of fatty acid synthase and lipoprotein lipase (*p* ˂ 0.05). Thus, it could be concluded that ducks fed on soybean hulls containing a diet at the level of 4.5% and providing 3.8% fiber level with or without lysolecithin showed the best performance.

## 1. Introduction

Duck farms in most developing countries are generally confined to smallholder farmers. This is mainly attributed to the lower availability of sufficient amounts of feed. Thus, searching for and introducing untraditional local feed ingredients in ducks’ diets has become of major concern. However, low-priced feedstuffs such as rice bran and seed hulls are generally low in energy and have a high dietary fiber content. Dietary fiber (DF), as a fraction of carbohydrate, is considered as an anti-nutritional factor in the poultry diet [1] which negatively affects feed palatability, feed intake (FI), and the digestibility of nutrients [2]. However, some previous trials summarized that poultry can tolerate moderate amounts of fiber in their diets which could help in the development of digestive system [3,4] and stimulate the production of gastric and bile acids (as well as endogenous enzymes) [5]. These effects might have a role in improving growth performance and gut health [6]. Moreover, Han, et al. [7], reported that supplementation of nanocrystalline cellulose improved the body gain and FI of ducks. Inconsistency in the results obtained between trials when investigating the impact of dietary fiber in poultry nutrition could be associated with differences in fiber type and diet formulation [8].

Lipid sources are used in poultry diets as supplemental energy sources to support their energy requirements. These sources are insoluble in the water medium of the poultry gut and need to be emulsified before digestion by lipolytic agents [9]. Young birds are inefficient in digestion and absorption of high levels of dietary lipids due to the limited secretion of bile salts and lipase before development of the gastro intestinal tract [10,11]. Therefore, supplementation of emulsifying agents or biosurfactants as lecithin and its derivative, lysolecithins could effectively help in improving fat utilization [12].

Lecithin is a by-product produced from the processing of vegetable oils with phospholipids as their main constituents [13]. As a derivative product, lysolecithins are formed by an enzymatic conversion of lecithin as the phospholipase enzyme removes one of the fatty acids from the phospholipids, producing lysophospholipid [14]. Lysophospholipid possesses more hydrophilic characteristics than lecithin, has better oil-in-water emulsifying properties [14,15], and thus is more able to promote the digestion of lipids. Previous reports documented that lysolecithin supplementation improves broiler growth performance [11,16]. Moreover, [17,18] reported that lysophospholipid supplementation to reduced-energy diets positively affected productive performance, nutrient utilization, and intestinal morphology in broiler chickens.

Limited information is available on the effects of crude fiber and emulsifying agents on Pekin duck performance. Utilization of nutrients, especially fat, may be improved with the addition of lysolecithin to the diet rather than emphasizing fiber as being an alternate feed ingredient. Perhaps lysolecithin can reduce the deleterious effects of high levels of fiber in the diet of young ducklings through helping in improving the efficiency of the utilization of the increased supplemental fat used. Therefore, this trial investigated the influence of dietary inclusion of soybean hulls as a source of DF and lysolecithin supplementation on performance, intestinal morphology, serum lipid profile, immune response, and gene expression of some lipid regulating genes of Pekin ducks.

## 2. Material and Methods

### 2.1. Duck’s Care and Experimental Design

Four hundred and eight unsexed one-day-old meat ducks (Pekin ducks) were individually weighed at the beginning of trial and then randomly allotted into eight equal groups. Each group was divided into 3 replicates with 17 bird/replicate. In a completely randomized design, eight diets with four concentrations of DF [2.4 (control diet), 3.8, 5.3, and 6.7%] supplemented in each case with or without 0.05% lysolecithin were offered. The dietary DF increments were achieved with the inclusion of soybean hulls at 0.0, 4.5, 9.0, and 13.5% of diet, respectively.

The control diets were formulated according to the NRC [19]. Ingredient composition of the diets and their chemical analysis according to the AOAC [20], are presented in Table 1. The lysolecithin source used in this experiment was Lysoforte (Kemin, animal health and Nutrition, Herentals, Belgium) which contains soya lecithin 50% with a carrier as silicic acid 30% and limestone up to 100%, with inclusion level of 0.05%. Growing ducks were housed in a clean well-ventilated room that was provided with heaters to maintain the ambient temperature according to the age of the ducks. Ducks were raised at room temperature initially set at 33 °C, then gradually reduced to 24–26 °C at the third week and exposed to 24 h constant light. Ducks were kept on floor pens bedded with wood shavings and provided with drinkers and feeders. Feed (mash form) and water were supplied ad libitum for the two-month experimental period. 

### 2.2. Growth Performance

Body weight (BW) and feed intake (FI) for each group were recorded biweekly. Performance indices were calculated as following: weight gain (WG) = final BW− initial BW; feed conversion ratio (FCR) = feed intake (g)/body gain (g); lipid efficiency ratio (LER) = weight gain (g)/lipid intake (g) and efficiency of energy utilization (EEU) = metabolizable energy intake (kcal)/body gain(g).

### 2.3. Serum Lipid Profile and Immune Response

At the end of the experiment and after 6 h of feed withdrawal, two ducks from each replicate were selected to collect the blood from the jugular vein. Two blood samples were collected (first in a citrated tube and the other without anticoagulant). The blood samples without anticoagulants were centrifuged at 3000 rpm for fifteen min. The serum was then isolated and stored at –20 °C until used for biochemical analysis. Serum lipid profile including triglyceride (TG), total cholesterol (TC), and low- and high-density lipoprotein (LDL and HDL) concentrations, in addition to activities of glutathione peroxidase (GPx) and catalase (CAT) and total antioxidant capacity (TAC) which were assessed using specific commercial kits (Biodiagnostic Co, Giza, Egypt). Immune response was evaluated by a group of parameters including phagocytic index and activity [21,22], lysozyme activity, and bactericidal activity. Activity of lysozyme was measured with the turbidimetric method described by Engstad, et al. [23] along with the bactericidal activity according to Rainger and Rowley [24]. 

### 2.4. Intestinal Morphology

After blood collection, birds were dissected and about 2.5 cm segment from the duodenum was trimmed and used for morphological indices. Samples of tissue were washed with saline then fixed in formalin 10% for 48 hrs. Slides were prepared and routinely stained with hematoxylin and eosin (H&E) according to Bancroft et al. [25]. The histomorphometric analysis was performed using Image J analysis software (National Institutes of Health, Bethesda, MD, USA). 

### 2.5. Expression Analysis of Genes Related to Lipid Metabolism

Liver samples (*n* = 4) from each treatment were taken into clean Eppendorf tubes and directly to liquid nitrogen then stored at –80 °C. Total RNA was extracted using TRIsure™ Kit (Sensi-Fast LO-ROX kit, Bioline, #94002. UK). The quality of RNA was verified on 1.5% agarose gel electrophoresis. The RNA samples were reverse transcribed to cDNA using the SensiFAST™ cDNA synthesis kit (Bioline, United Kingdom).

As presented in Table 2, primer sequences were used to amplify fatty acid synthetase (*FAS*) and lipoprotein lipase (LPL) according to Jiang, et al. [26]. Real-time was performed in Stratagene MX300P Real-time PCR (Agilent Technologies, Santa Clara, USA) machine, using the SensiFast™ SYBR Lo-Rox kit (Bioline, United Kingdom) following the manufacturer’s guidelines. The PCR protocol was as following: initial denaturation at 95 °C for fifteen min, followed by 40 cycles at 95 °C for 15 s, and 60 °C for 1 min. Melting curve analyses were run to ensure a single product of each reaction. The values of Ct of the target genes were first normalized against Ct of the house-keeping gene (β-actin). These were then used to calculate the relative gene expression of the target gene as a fold change based on the Livak Method [27], where fold change equal 2^−∆∆CT^.

### 2.6. Statistical Analysis

The obtained results were analyzed by two-way ANOVA to investigate the impacts of different fiber levels, lecithin, and their interaction. 

The statistical model used was:

Y_ijk_= μ+ A_i_ + S_j_ + AS_ij_ + e_ijk_,

where Y_ijk_ is an observation, μ is the overall mean, A_i_ is effect of fiber level (I = 1–4), S_j_ is effect of lysolecithin level (j = 1–2), AS_ji_ the interactions between two variables, and e_ijk_ is the experimental random error. The post-hoc Tukey’s test was carried out to detect differences among treatments. All differences were considered to be significant at *p* < 0.05.

## 3. Results

### 3.1. Performance and Feed Efficiency Parameters

**Figure 1:**
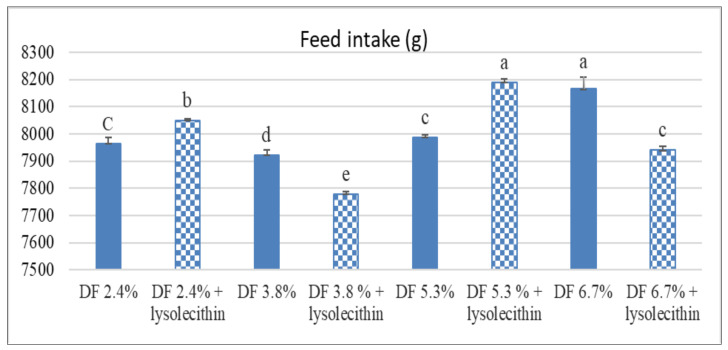
Effect of different fiber levels with or without lysolecithin supplementation on feed intake (g) of growing pekin ducks.

As shown in Table 3, final BW and WG were not affected by DF levels, lysolecithin supplementation, and the interaction between them (*p* > 0.05). Increasing DF% to 6.7% through the inclusion of soybean hulls in diet non-significantly reduced duck BW (*p* > 0.05). However, feeding on 3.8% DF exhibited the highest BW. DF level (5.3%) with lysolecithin significantly improved BW (*p* < 0.05) compared with the highest level of DF (6.7%) which reduced it. Moreover, duck’s FI was significantly affected by DF levels and the interaction between it and lysolecithin added (*p* < 0.05) as shown in Figure 1. The FI was reduced in ducks received diets contain 3.8% with or without lysolecithin addition, while increased with increasing fiber level in the diet compared to control. Regarding the feed efficiency utilization, the effect of different DF levels was extended to FCR and EEU as it was improved in ducks fed diet contains 3.8% DF while were deteriorated with increasing DF level with or without lysolecithin. On the other hand, lysolecithin addition significantly affected (*p* ˂ 0.05) the lipid efficiency ratio (LER) as it was reduced in those supplemented with lysolecithin when compared with their relative control groups. Ducks fed soybean hulls providing fiber level 3.8% showed improved average FCR and EEU when compared with the other groups. LER was gradually decreased with increasing dietary fiber levels (*p* < 0.001), but it was not affected by the main effect of lysolecithin or the interaction between DF and lysolecithin (*p* > 0.05).

### 3.2. Immune Response, Antioxidants and Serum Lipid Profile

As presented in Table 4, DF levels and lysolecithin supplementation significantly affected the immune response parameters including phagocytic activity and index (PA and PI) (*p* < 0.05). These parameters were significantly improved with increasing fiber concentration in diet, with the highest being in those supplemented with lysolecithin. On the other hand, lysosomal activity was modified by different fiber levels included in the diet as it was enhanced with increasing DF% compared with G1 (received 2.4%), with the highest activity in G2 (received 3.8%). Moreover, lysolecithin addition altered the bactericidal activity as it was reduced in ducks supplemented with lysolecithin in their diet. Moreover, increasing fiber level and lysolecithin inclusion affected the serum concentrations of CAT and GPx (*p* ˂ 0.05) (Table 5). Serum CAT and GPx activities with increasing the fiber level in the diet with or without lysolecithin were increased when compared with control. The same trend was obtained with serum TAC, as it showed non-significant increase (*p* > 0.05). Regarding the serum lipid profile presented in Table 6, increasing DF% in the diet with or without lysolecithin addition had no significant effect on the serum concentrations of lipid profile parameters (triglyceride, total cholesterol, low density lipoprotein, very low-density lipoprotein) (*p* > 0.05). However, serum HDL concentration was significantly altered by the DF levels as it was increased with increasing fiber level in diet (*p* ˂ 0.05).

### 3.3. Intestinal Morphology

**Figure 2:**
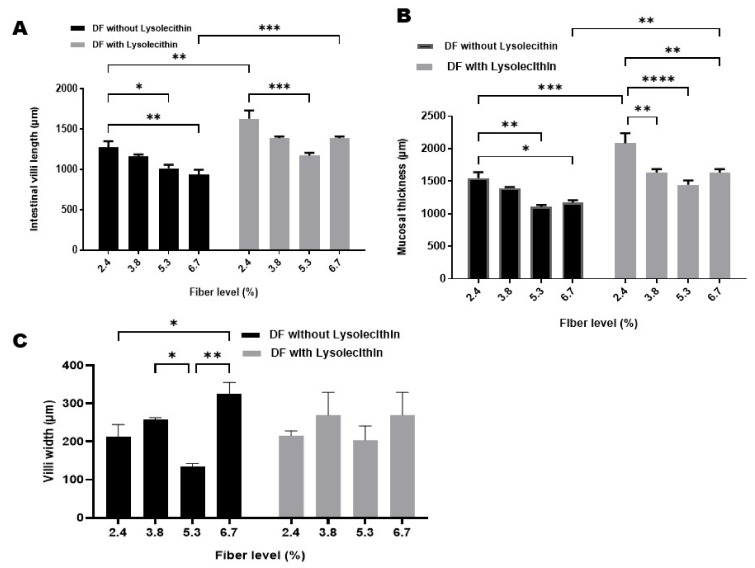
Intestinal morphology of growing Pekin ducks fed on different fiber levels with or without lysolecithin supplementation. (**A**) represents the intestinal villi length, (**B**) represents the mucosal thickness, and (**C**) represents the intestinal villi width. Results are expressed as mean ± SEM. * *p* < 0.05; ** *p* < 0.01; *** *p* < 0.001; **** *p* < 0.0001.

**Figure 3:**
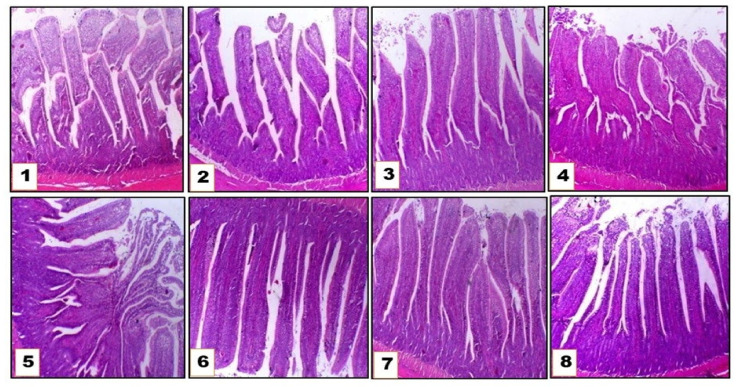
Photomicrograph of intestinal morphology (duodenum) of growing Pekin ducks fed on different fiber levels with or without lysolecithin supplementation (H&E).

As presented in Figure 2 and Figure 3, different fiber levels and lysolecithin addition altered the duodenum morphology in terms of intestinal villi length, width, and mucosal layer thickness. Increasing DF up to 6.7% through soya hulls inclusion in duck’s diet reduced (*p* ˂ 0.05) the duodenal villi length and mucosal layer thickness, while increasing villi width (*p* ˂ 0.05) when compared with the control group who received 2.4% DF. Lysolecithin supplementation with different DF levels alleviated these adverse effects of increasing fiber level on duodenum morphology, as it increased villi length and mucosal layer thickness compared with control groups without lysolecithin addition.

### 3.4. Expression of Some Fat Metabolism-Regulating Genes in Duck Liver

**Figure 4:**
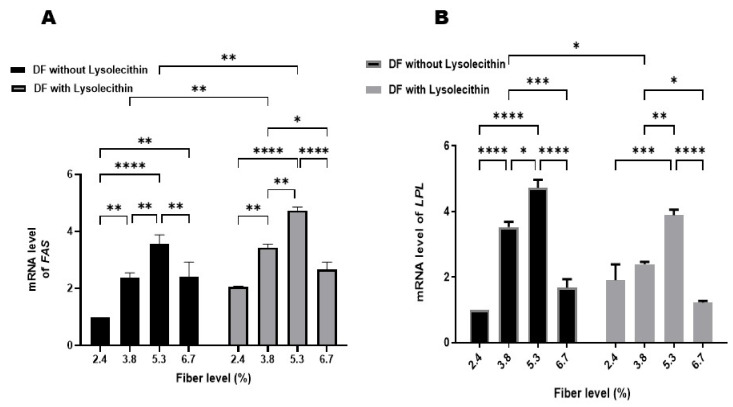
Hepatic mRNA level of fatty acid synthase (*FAS*, **A**) and lipoprotein lipase (LPL, **B**) in growing Pekin ducks fed on different fiber levels with or without lysolecithin supplementation. * *p* < 0.05; ** *p* < 0.01; *** *p* < 0.001; **** *p* < 0.0001.

Different fiber levels and lysolecithin supplementation significantly modulated the gene expression of *FAS* and LPL (*p* ˂ 0.05) as presented in Figure 4. Increasing DF level in ducks’ diets with or without lysolecithin significantly induced a distinct increase in the expression of both studied genes compared with their control groups with the highest expression noticed in ducks fed on diets contain 5.3% DF. A dietary fiber level of 6.7% in the diet increased the relative abundance of mRNA of *FAS* and LPL compared with the control group received 2.4% DF. However, it was lowered when compared with other DF levels included in the diet (3.8 and 5.3%).

## 4. Discussion

Despite its importance as a nutrient, fiber level in the poultry diet should be taken into consideration as it has limited ability to digest it and increasing levels could have adverse effects. The present study showed that increasing fiber levels (3.8, 5.3, and 6.7%) in the ducks’ diets resulted in a non-significant reduction in their BW. Ducks fed on 3.8% DF demonstrated heavier BW which suggests that this amount of fiber is adequate for duck performance while higher levels interfere with nutrient digestion and absorption, resulting in lower performance. In the same line, Tejeda and Kim [28] found that broilers fed on 4% soybean hulls containing diet had the highest WG while higher levels (6 and 8%) and reduced birds’ WG. Also, Sadeghi et al. [29] found that 3% fiber from dietary sugar beet pulp and rice hulls reduced the daily WG in broiler chicks from 1 to 14 days. On the contrary, Abd El-latif [30] reported that BW was non-significantly affected by feeding Pekin ducks on diets containing DF up to 12% at two to eight weeks of age. Additionally, Beshara, et al. [31], reported no difference in BW of growing ducks during 6–18 weeks of age fed different DF (3.65, 4.77, or 6.23%). A possible explanation for the inconsistent results between studies could be associated with species difference (chick vs duck), fiber source included in the diet, and the fact that meat ducks have a high tolerance to fiber than broilers [28,32]. Ducks fed on diets containing 3.8% DF showed the best performance while higher levels had negative impacts on these performance parameters. The obtained result of increased FI with increasing fiber level could be related to those birds trying to compensate for the lower nutrient concentrations in the fibrous-containing diet by increasing the feed consumption. This increased FI with the lower body gain negatively affected the feed efficiency utilization as reported by deteriorated FCR and PER with increasing fiber level inclusion. In support, Han et al. [32], showed that increasing DF% increased FI and deteriorated FCR of ducks. 

As far we know, no previous studies dealing with the effect of lysolecithin supplementation with different DF concentrations on ducks BW. In the current study, lysolecithin supplementation had no effect on BW and the gain of Pekin ducks fed on diets contains higher DF levels. Moreover, the interaction between the two main factors affected ducks’ FI, as it was reduced when lysolecithin added to the DF containing diets (3.8 and 6.7%). In the same direction, Zosangpuii et al. [33] observed that the BW of ducks fed on soybean oil with an emulsifier was better at days 14 to 28. However, in the subsequent periods, BW was similar to other treatments. Unlike the obtained results, [11,12] documented that the addition of lysolecithin to broiler diets improved their growth performance and feed efficiency. Overall, the addition of lysolecithin with the gradual increased fiber level included in this study didn’t support growth performance traits of growing ducks, as this could be attributed to the increased level of supplemental vegetable oil included in diets, source of oil used, and the dose of lysolecithin used. In support, Jansen et al. [34] demonstrated that the improvements that can be made with lysolecithin supplementation are highly dependent on the fat incorporated in broiler feeds. Furthermore, Jansen et al. [34] reported that the beneficial properties of lysolecithin might be affected by its chemical composition, and source of lecithin from which the lysolecithin is derived. One more possible explanation for the lack of effect of lysolecithin addition with higher fiber levels is the source of the fiber which could contain antinutritional factors negatively affect the intestinal viscosity and gut motility and consequently impact the action of the emulsifier added. As shown in our results, the dietary addition of lysolecithin was more effective with diets containing a 3.8% fiber level compared with the higher levels of fiber included, which could negatively impact the digestibility and utilization of supplemental fat used. This could be supported by the lipid efficiency ratio in this study, as increasing the fiber level in diet reduced it.

The immune response of ducks was enhanced with different DF levels (either single or combined with lysolecithin supplementation). This response may be attributed to the higher percentage of pectin of soybean hulls, which acts as prebiotics that could have an immunostimulanting effect. The present data suggest that growing Pekin ducks may prefer extra fiber concentration to enhance their immune response. This finding is in line with Sadeghi, et al. [29] who found that broiler feeding on sugar beet pulp/rice hulls increased (*p* < 0.05) antibody titer against Newcastle disease vaccine (NDV). Similarly, Abou-Elkhair, et al. [35] stated that the inclusion of both lysoforte (lecithin-containing product) and yeast in broiler diets contain a dry fat improved antibody titer against NDV. Lysophosphatidylcholine plays an essential role in the activation of T- lymphocyte and improves the humoral and cellular immunity of broiler chickens [36]. Moreover, Lysophosphatidylcholine activates monocytes and macrophages consequently causing improve phagocytosis of mice [37]. In addition, high DF and lysolecithin supplementation enhanced serum antioxidant enzyme activities. In support, Behera, et al. [38] found that increasing level of citrus waste (a source of soluble fiber) in the broiler diet improved (*p* < 0.05) antioxidant activities (SOD). Soya lecithin is considered a strong antioxidant due to its phospholipids content [39]; as a lecithin derivative, lysolecithin stimulated the antioxidants activity.

Serum lipid profile parameters including TG, LDL, and HDL are considered as the main indicators of lipid metabolism [40]. In the present study, DF% with or without lysolecithin non-significantly reduced TG and LDL and increased HDL serum concentrations. The obtained findings are in line with Beshara et al. [31], who found that HDL was slightly higher, with lower TG level for ducks fed different levels of CF. Qin, et al. [41], however, stated that higher DF% decreased serum TC and had no significant effect TG, HDL or LDL concentrations in growing ducklings. DF reduced serum LDL and increased HDL [42,43] as fiber interferes with lipid absorption and metabolism through binding to bile acid and cholesterol, consequently lowering serum lipids [44]. Therefore, it can be concluded that a DF% more than 2.4% may have an important role in modulating the serum lipid profile of meat ducks. The addition of lysolecithin to the control group containing 2.4% fiber level induced a lowering effect on serum TC and LDL concentrations in this experiment. Similarly, Zosangpuii et al. [33] concluded that serum TC was reduced (*p* > 0.05) with the emulsifier fed ducks compared with control. This response of reduced TC with lysolecithin addition may be returned so that it reduces the absorption of cholesterol and increases its excretion [45,46]. On the other hand, Hu et al. [47] found that the addition of emulsifier (Aldo) in ducks’ diet replacing two different oils reduced TG and increased the activity of lipoprotein and hepatic lipases in liver and of pancreatic lipase (while having no effect on serum TC, LDL, or HDL). Moreover, lysolecithin addition with increased DF% containing diets did not affect lipid profile parameters. Likewise, Park et al. [16] stated that dietary exogenous lysolecithin supplementation didn’t alter serum TC, TG, or free fatty acid concentrations in broilers. It could be summarized that lipid metabolites in ducks depend on diet composition and emulsifier type. Generally, previous studies on the effect of lysolecithin on serum lipid profiles of growing ducklings are very limited. Further investigation on the use of this biosurfactant in modulating lipid metabolism in ducks could be required.

Dietary fiber affects intestinal morphology and absorptive role [48]. In the current study, high DF in ducks’ diets decreased villi length and mucosal thickness. Similarly, Tejeda and Kim [28] found that feeding on soybean hull (6 or 8%) reduced the villus height in the jejunum and ileum, and Ling et al. [49] reported that higher fiber levels in geese ration reduced jejunal villi length and reduced duodenal villi height of turkey with high DF [1]. This reported effect on the intestinal morphology could be attributed to the abrasive effect of the fiber on the mucosal surface of the intestine, shortening of the intestinal villi, and consequent reducing of the absorptive area and nutrient absorption, which could explain the lower performance obtained in ducks with the gradual increase in fiber level. On the contrary, Han et al. [32] observed that ducks fed a 7.52% DF diet had increased (*p* < 0.05) mucosal layer thickness compared to ducks fed 1.46, 3.09, and 9.03% DF containing diets. This difference may be related to variations in fiber source used in each trial, differences in gastrointestinal structure between meat ducks and broiler, and adaptability of duck to higher DF than broiler chicks. Lysolecithin supplementation reduced the negative effects of increasing fiber level on duodenum morphology. This improvement in villi length and width is in line with [17] who found that dietary lysophospholipid (LPL) supplementation increased (*p* < 0.05) jejunal villi length of broiler chickens. Also consistent with [18] who found that villus height (VH) and VH, crypt depth (CD), both in the jejunum and duodenum, were significantly increased in broilers fed with lysolecithin or/and xylanase supplemented diet.

The LPL had an important catabolic role and is involved in the hydrolysis of TG producing free fatty acids and glycerol, while *FAS* is involved in lipogenesis [41] as the enzyme which catalyzes fatty acid synthesis. The increased expression of LPL in this study suggested that increased DF% plays an important role in lipid metabolism in meat ducks. The liberated fatty acids are then used as an energy source or storage in the adipose tissue, stimulating the expression of genes associated with these metabolic pathways. Furthermore, the relative mRNA of *FAS* was increased when increasing the DF% level in diets, with higher expression noted in the lysolecithin supplemented groups compared with the same ducks which received a different DF% without lysolecithin addition. Similarly, Huang et al. [45] reported that soy lecithin upregulated the gene expression of *FAS*, acetyl-CoA carboxylase (ACC), and sterol regulatory element binding protein-1 (SREBP-1) in broilers. This is inconsistent with Qin et al. [41], who found downregulation of expression of the previously mentioned gene in the liver with increasing DF% in meat ducks’ diet, and an increase of the adipose triglyceride lipase. Furthermore, Hosseini et al. [18] reported that dietary supplementation of lysolecithin or/and xylanase did not have any effect on hepatic lipogenic gene expression (*FAS* and *L-FABP)* of broiler chickens fed on a low energy diet.

In general, DF is classified to soluble and insoluble DF (SDF and IDF, respectively), SDF affect the lipid profile through reducing lipid and cholesterol level, while the IDF stays in the intestinal tract altering the composition of the gut microbiota and their fermentation process, and consequently affecting the short chain fatty acids (SCFA) produced [50]. The variation in obtained results between trials could be attributed to the DF type and amount, species under the study, and the microflora in the gut. Therefore, the alteration in the gene expression in this study could be associated with modulation of intestinal flora caused by fiber in soya hulls and subsequently affecting SCFA production. Limitation in the number of genes investigated in the current trial provided an incomplete vision about the effects of our treatments on lipid metabolism. Thus, further investigations are required to understand the assumption behind the regulatory effect of crude fiber soya hulls on lipid metabolism through studying the expression levels of more genes involved in lipolysis and lipogenesis as well as their effect on cecal microflora fermentation.

## 5. Conclusions

Based on the previously mentioned results, it could be concluded that increasing fiber level in the diet of meat ducks through the inclusion of soya hulls with or without lysolecithin addition altered their live performance and intestinal morphology, improved immune response, and had a modulatory effect on the expression of some lipid metabolism-regulating genes. Our results showed that meat ducks fed on 4.5% soya hulls (3.8% fiber) exhibited the best growth performance among other dietary levels. Further studies are required for investigating the impact of soya hulls on cecal microflora profile, their fermentation products, and their regulatory effect on lipid metabolism.

## Figures and Tables

**Table 1:** Ingredients and chemical composition of diets.

Ingredients (%)	Starter				Grower-Finisher			
	D1	D2	D3	D4	D1	D2	D3	D4
Soybean hulls	0	4.5	9.0	13.5	0	4.5	9.0	13.5
Corn grain	58.95	53.7	48.7	44.15	68.45	63.2	58.45	53.95
Soybean meal	30.0	30.0	30.0	30.0	23.5	23.0	22.5	22.0
Corn gluten	7.0	6.5	6.25	5.8	4.0	4.0	4.0	4.0
Vegetable oil	0.5	1.75	2.5	3.0	0.5	1.75	2.5	3.0
Dicalcium phosphate ^1^	1.7	1.7	1.7	1.7	1.7	1.7	1.7	1.7
Limestone	0.9	0.9	0.9	0.9	0.9	0.9	0.9	0.9
Premix ^2^	0.3	0.3	0.3	0.3	0.3	0.3	0.3	0.3
Toxin Binder ^3^	0.05	0.05	0.05	0.05	0.05	0.05	0.05	0.05
Anticolostridi ^4^	0.05	0.05	0.05	0.05	0.05	0.05	0.05	0.05
Choline ^5^	0.1	0.1	0.1	0.1	0.1	0.1	0.1	0.1
Lysine ^6^	0.1	0.1	0.1	0.1	0.1	0.1	0.1	0.1
Methionine	0.1	0.1	0.1	0.1	0.1	0.1	0.1	0.1
Salt	0.25	0.25	0.25	0.25	0.25	0.25	0.25	0.25
Chemical composition (%)								
Crude protein	21.98	21.84	21.87	21.80	18.04	17.97	17.95	17.94
Ether extract	3.27	4.35	4.96	5.33	3.48	4.57	5.18	5.56
Crude fiber	2.40	3.85	5.31	6.78	2.36	3.79	5.24	6.69
Calcium	0.89	0.91	0.93	0.95	0.88	0.89	0.91	0.93
Phosphorus	0.37	0.37	0.37	0.37	0.36	0.36	0.36	0.35
Lysine	1.10	1.12	1.13	1.14	0.93	0.93	0.94	0.95
Methionine	0.49	0.48	0.47	0.46	0.43	0.42	0.41	0.40
Threonine	0.82	0.81	0.81	0.80	0.68	0.67	0.67	0.66
Metabolizable Energy, kcal/kg	2981.7	2937.75	2913.95	2897.0	3003.4	3006.9	2989.6	2961.9
NDF *	10.93	13.38	15.88	18.41	10.47	12.91	15.41	17.92

^1^ Greenphos Dicalcium 18%: produced by Adana Company, Turkey, composed of Phosphorus 18%; Calcium 25% and Fluorine 0.18%). ^2^ HY- Mix Min Broiler duck: Premix produced by Misr Feed Additives Company, Egypt and composed of (Retinyl acetate 15000000 IU; Cholecalciferol 3500000 IU; Tocopherol acetate 25000 mg; Menadione nicotinamide 3000 mg; Thiamine mononitrate 2000 mg; Riboflavin 10000 mg; Pyridoxine HCL 5000 mg; Cyanocobalamin 20 mg; Pantothenic 15000 mg; Niacin 45000 mg; Folic acid 1500 mg; Biotin 100 mg; choline Chloride 800 g; Manganese 120 g; Zinc 80 g; Iron 60 g; Copper 6 g; Iodine 0.50 g; Selenium 0.30 g and Cobalt 0.10 g. ^3^ Fixfin produced by Kemin Belgium, composed of Bentonite-montmorillionate,55.6%; Sepiolite 44.4%. ^4^ Clostat: Clostat. produced by Kemin Belgium, composed of Bacillus Subtilis spores 0.2%; Maltodextrin 0.8% and Calcium Carbonate up to 100%. ^5^ Choline Chloride: produced by Liaoning Biochem. Co., Ltd. ^6^ L-Lysine Monohydrochloride: produced by PT-Cheil Jedang Indonesia. * NDF (neutral detergent fiber) was calculated.

**Table 2:** Sequence of forward and reverse primers used in real Time PCR.

Gene	Primer Sequence	Accession
*FAS* ^1^	F: GCTGAGAAACGCCAATACC R: GAGCAAGACACCGCAAACT	NM_001310798.1
B-actin	F: GGTATCGGCAGCAGTCTTA R: TTCACAGAGGCGAGTAACTT	NM_00131042.1
LPL ^2^	F: AAGAGGGAACCTGATTCAAACG R: CCATCCAGTCAATAAACATAGCG	FJ859348.1

^1^*FAS*: fatty acid synthetase, ^2^ LPL: lipoprotein lipase.

**Table 3:** Growth performance of growing Pekin ducks fed on different fiber levels with or without lysolecithin supplementation (*n* = 3).

Item	Initial BW (g)	Final BW (g)	Total BW Gain (g)	FI (g)	FCR (g Feed/g Gain)	LER (g Gain/Lipid Intake)	EEU (ME Intake/g Gain)
Fiber Effect (%)							
2.4	54.500	2531.300	2476.800	8007.325 ^b^	3.270 ^ab^	9.097 ^a^	9.794 ^ab^
3.8	54.031	2541.837	2487.806	7850.643 ^c^	3.187 ^b^	7.062 ^b^	9.558 ^b^
5.3	54.350	2545.000	2490.650	8088.150 ^a^	3.286 ^ab^	6.042 ^c^	9.796 ^ab^
6.7	54.050	2460.500	2406.450	8052.800 ^a^	3.392 ^a^	5.461 ^d^	10.025 ^a^
SEM	0.440	27.280	26.922	14.070	0.036	0.075	0.107
Lysolecithin effect (%)							
0	54.343	2546.104	2491.761	8009.973	3.248	6.987	9.688
0.05	54.122	2493.214	2439.092	7989.486	3.319	6.845	9.897
SEM	0.136	19.344	19.085	9.974	0.025	0.053	0.76
Two-way Anova (*p*-value)							
DF level	0.893	0.09	0.09	<0.001	0.001	<0.001	0.025
Lysolecithin	0.621	0.054	0.052	0.147	0.050	0.060	0.051
Interaction	0.107	0.105	0.090	0.001	0.841	0.385	0.849

Means in the same column within each classification bearing different letters are significantly (*p* ≤ 0.05) different. SEM: standard error of mean. FI: feed intake; FCR: feed conversion ratio; LER: lipid efficiency ratio; EEU: efficiency of energy utilization.

**Table 4:** Immune response of growing Pekin ducks fed on different fiber levels with or without lysolecithin supplementation (*n* = 6).

Item		Phagocytic Activity	Phagocytic Index	Lysozyme Activity	Bactericidal Activity
Fiber Effect (%)					
2.4		44.533 ^c^	1.151 ^b^	0.405 ^b^	58.664
3.8		46.721 ^b^	1.365 ^ab^	0.766 ^a^	62.863
5.3		47.623 ^b^	1.329 ^b^	0.670 ^a^	65.063
6.7		49.273 ^a^	1.579 ^a^	0.649 ^a^	63.375
SEM		0.483	0.067	0.051	1.809
Lysolecithin effect (%)					
0		45.668	1.229	0.579	57.682
0.05		48.406	1.483	0.666	67.300
SEM		0.342	0.047	0.036	1.279
Two-way Anova (*p*-value)					
DF level		<0.001	0.002	<0.001	0.107
Lysolecithin		0.001	0.107	<0.001	0.951
Interaction		0.389	0.126	0.093	0.404

Means in the same column within each classification bearing different letters are significantly (*p* ≤ 0.05) different. SEM: standard error of mean.

**Table 5:** Serum antioxidants in growing Pekin ducks fed on different fiber levels with or without lysolecithin supplementation (*n* = 6).

Item	Total Antioxidant Capacity (TAC, u/mL)	Glutathione Peroxidase (GPx, u/mL)	Catalase (u/mL)
Fiber Effect (%)			
2.4	18.369 ^c^	205.838	1.021
3.8	26.088 ^b^	270.763	1.138
5.3	26.513 ^b^	279.488	1.178
6.7	32.063 ^a^	309.700	1.188
SEM	3.106	28.405	0.048
Lysolecithin effect (%)			
0	22.153	231.638	1.090
0.05	29.363	301.256	1.172
SEM	2.197	20.085	0.034
Two-way Anova (*p*-value)			
DF level	0.038	0.096	0.080
Lysolecithin	0.029	0.022	0.100
Interaction	0.305	0.985	0.738

Means in the same column within each classification bearing different letters are significantly (*p* ≤ 0.05) different. SEM: standard error of mean.

**Table 6:** Serum lipid profile of growing Pekin ducks fed on different fiber levels with or without lysolecithin supplementation (*n* = 6).

Item	Triglyceride (mg/dL)	Total Cholesterol (mg/dL)	High-Density Lipoprotein (HDL, mg/dL)	Low-Density Lipoprotein (LDL, mg/dL)	Very Low-Density Lipoprotein (VLDL, mg/dL)
Fiber Effect (%)					
2.4	200.288	206.526	50.436 ^c^	116.033	40.058
3.8	199.505	206.076	52.778 ^b^	113.398	39.901
5.3	198.960	206.639	53.420 ^b^	113.427	39.792
6.7	195.958	205.749	54.566 ^a^	111.991	39.192
SEM	1.227	0.889	0.887	1.337	0.245
Lysolecithin effect (%)					
0	198.639	206.570	52.469	114.373	39.728
0.05	198.716	205.925	53.131	113.051	39.743
SEM	0.868	0.629	0.627	0.945	0.174
Two-way Anova (*p*-value)					
DF level	0.095	0.885	0.022	0.218	0.095
Lysolecithin	0.951	0.475	0.462	0.332	0.951
Interaction	0.663	0.262	0.091	0.086	0.636

Means in the same column within each classification bearing different letters are significantly (*p* ≤ 0.05) different. SEM: standard error of mean.
